# A Systematic Analysis on DNA Methylation and the Expression of Both mRNA and microRNA in Bladder Cancer

**DOI:** 10.1371/journal.pone.0028223

**Published:** 2011-11-30

**Authors:** Jialou Zhu, Zhimao Jiang, Fei Gao, Xueda Hu, Liang Zhou, Jiahao Chen, Huijuan Luo, Jihua Sun, Song Wu, Yonghua Han, Guangliang Yin, Maoshan Chen, Zujing Han, Xianxin Li, Yi Huang, Weixing Zhang, Fangjian Zhou, Tong Chen, Pingping Fa, Yong Wang, Liang Sun, Huimin Leng, Fenghao Sun, Yuchen Liu, Mingzhi Ye, Huanming Yang, Zhiming Cai, Yaoting Gui, Xiuqing Zhang

**Affiliations:** 1 Beijing Genomics Institute at Shenzhen, Shenzhen, China; 2 College of Life Science, Wuhan University, Wuhan, China; 3 Guangdong and Shenzhen Key Laboratory of Male Reproductive Medicine and Genetics, Institute of Urology, Peking University Shenzhen Hospital, Shenzhen PKU-HKUST Medical Center, Shenzhen, China; 4 Beijing Institute of Genomics, Chinese Academy of Sciences, Beijing, China; 5 Graduate University of Chinese Academy of Sciences, Beijing, China; 6 Department of Urology, The Second People's Hospital of Shenzhen, Shenzhen, China; 7 Department of Urology, the First Affiliated Hospital of Zhengzhou University, Zhengzhou, China; 8 Department of Urology, Sun Yat-Sen University Cancer Center, Guangzhou, China; 9 Department of Urology, Shenzhen People's Hospital, Shenzhen, China; 10 Shantou University Medical College, Shantou, China; University of Oklahoma and Oklahoma Medical Research Foundation, United States of America

## Abstract

**Background:**

DNA methylation aberration and microRNA (miRNA) deregulation have been observed in many types of cancers. A systematic study of methylome and transcriptome in bladder urothelial carcinoma has never been reported.

**Methodology/Principal Findings:**

The DNA methylation was profiled by modified methylation-specific digital karyotyping (MMSDK) and the expression of mRNAs and miRNAs was analyzed by digital gene expression (DGE) sequencing in tumors and matched normal adjacent tissues obtained from 9 bladder urothelial carcinoma patients. We found that a set of significantly enriched pathways disrupted in bladder urothelial carcinoma primarily related to “neurogenesis” and “cell differentiation” by integrated analysis of -omics data. Furthermore, we identified an intriguing collection of cancer-related genes that were deregulated at the levels of DNA methylation and mRNA expression, and we validated several of these genes (HIC1, SLIT2, RASAL1, and KRT17) by Bisulfite Sequencing PCR and Reverse Transcription qPCR in a panel of 33 bladder cancer samples.

**Conclusions/Significance:**

We characterized the profiles between methylome and transcriptome in bladder urothelial carcinoma, identified a set of significantly enriched key pathways, and screened four aberrantly methylated and expressed genes. Conclusively, our findings shed light on a new avenue for basic bladder cancer research.

## Introduction

As genetic and epigenetic abnormalities have been observed in the development of cancer cells [1], it has been theorized that they both play important roles in oncogenesis, inducing changes in gene activity and chromosome structure [2]. Basic research to reveal mechanisms of carcinogenesis is extremely important for the diagnosis, treatment and prognosis of cancers in the clinic. The aberration of DNA methylation in cancer can occur as global hypomethylation or locus-specific hypermethylation in CpG island-rich promoters [3]. DNA hypomethylation in a cancer cell is thought to lead to chromosome instability and oncogene activation [4]. Conversely, aberrant promoter methylation or hypermethylation of CpG islands-associated genes is strongly associated with transcriptional silencing of these genes [5]. In addition to DNA methylation, deregulated miRNAs in cancer may affect the expression of genes and pathways that are involved in cancer pathogenesis all the way from initiation to metastasis. Some miRNAs serve as putative tumor suppressors and others act as oncogenes [6].

Bladder cancer is one of the most common cancers in the world. In 2008, an estimated 386,300 new cases were diagnosed, and 150,200 people died from bladder cancer [7]. Urothelial carcinoma of the bladder, the most prevalent histological type of bladder cancer, makes up more than 90% of the malignant cases of the bladder cancer [8]. DNA hypomethylation is a common phenomenon in bladder cancer [9], [10]. Meanwhile, the relationship between DNA methylation and bladder cancer has primarily been studied with regard to promoter hypermethylation of tumor suppressor genes. Inappropriate silencing of these genes by promoter hypermethylation contributes to cancer initiation, progression, invasion, and metastasis [11], [12], [13]. A number of studies have examined both small and large panels of DNA methylation events in bladder cancer using array-based technologies [9], [10], [14]. However, genome-wide detection studies to obtain high-resolution methylation data in bladder cancer are rare. The tumor-suppressor and oncogenic functions of miRNAs have been observed in bladder cancer as well [15], [16], [17]. Several miRNA profilings by microarrays in bladder cancer have also been carried out, but no consistent conclusion could be always drawn from their results [18], [19], [20].

Based on above observations, we hypothesized that a systematic analysis on profiles of DNA methylation and expression of both mRNA and microRNA in bladder cancer would help to identify key alterations of bladder cancer. Thus, we carried out a genome-wide methylation analysis in matched tumor and normal adjacent tissues from 9 bladder urothelial carcinoma patients using modified methylation-specific digital karyotyping (MMSDK), combined with mRNA and miRNA expression data (coupled with the results of our previous studies on mRNA [21] and miRNA [22] of bladder cancer), which was obtained from tag sequencing using second-generation sequencing technology.

## Results

### An overview of methylome and transcriptome in bladder urothelial carcinoma

For DNA methylation, an average of 4,445,972 and 4,525,269 uniquely mapped tags that were 17 and 18 nt in length were obtained from 9 different bladder urothelial carcinomas and their matched normal adjacent tissues, respectively. For gene expression, the DGE libraries generated 3,307,536 and 3,136,370 high-quality tags for tumor and normal tissues, respectively. In non-coding small RNA (sRNAs) sequencing, a total of 14,787,388 and 12,754,928 high-quality reads were obtained for tumor and normal tissues, respectively. In total, 16,236 genes and 543 miRNAs were detected expressed in at least one of the tumor or normal adjacent tissue samples.

**Figure 1:**
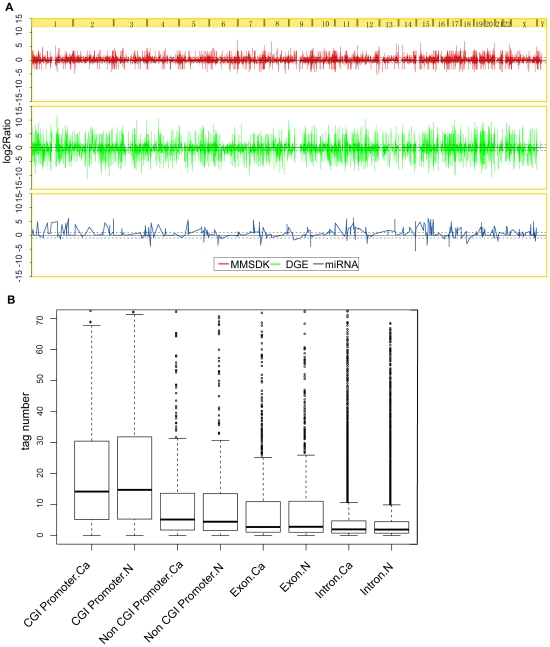
Overview of DNA methylation patterns in relation to gene expression. A. Genome wide DNA methylation, mRNA, and miRNA profiles from bladder cancer tissues and matched normal adjacent tissues. The log2-transformed average fold changes (tumor/normal) for methylation status (red), mRNA expression level (green), and miRNA expression level (blue) from all patients were drawn across the whole genome. The y-axis represents the log2 ratio, and the dashed lines represent the significance threshold (|log2Ratio| = 1). B. Distribution of the methylation levels in different types of genic regions between bladder cancer tissues and matched normal adjacent tissues. The box-plots for the distribution of DNA methylation levels based on the log2-transformed tag fold change (tumor/normal) from MMSDK across all patients were drawn. Types include CpG-rich promoters, CpG-poor promoters, exons, and introns.

To obtain an overview of the differences in the DNA methylation patterns with regard to the gene expression profiles in the bladder urothelial carcinomas and their matched normal adjacent tissues, the log2-transformed, average-fold changes between tumor and normal tissues for the three types of tags were plotted for the entire genome (Figure 1A). At a threshold of |log2Ratio|≥1, we observed some differences in the tags that represent the methylation status at certain *MluI* genomic loci and the mRNA expression level between cancer tissues and normal controls, Furthermore, a global up-regulation of miRNAs in bladder cancer was observed.

To further examine the methylation status at different genomic regions, we compared the methylation profiles of promoters with or without CpG islands (CGIs), exons and introns of bladder urothelial carcinoma tissues and the matched normal adjacent tissues. A similar level of overall DNA methylation in the examined genomic regions was distributed between two types of tissues (Figure 1B, P>0.05, Wilcoxon rank-sum test).

### Identification of divergent loci of DNA methylation and differentially expressed genes

**Figure 2:**
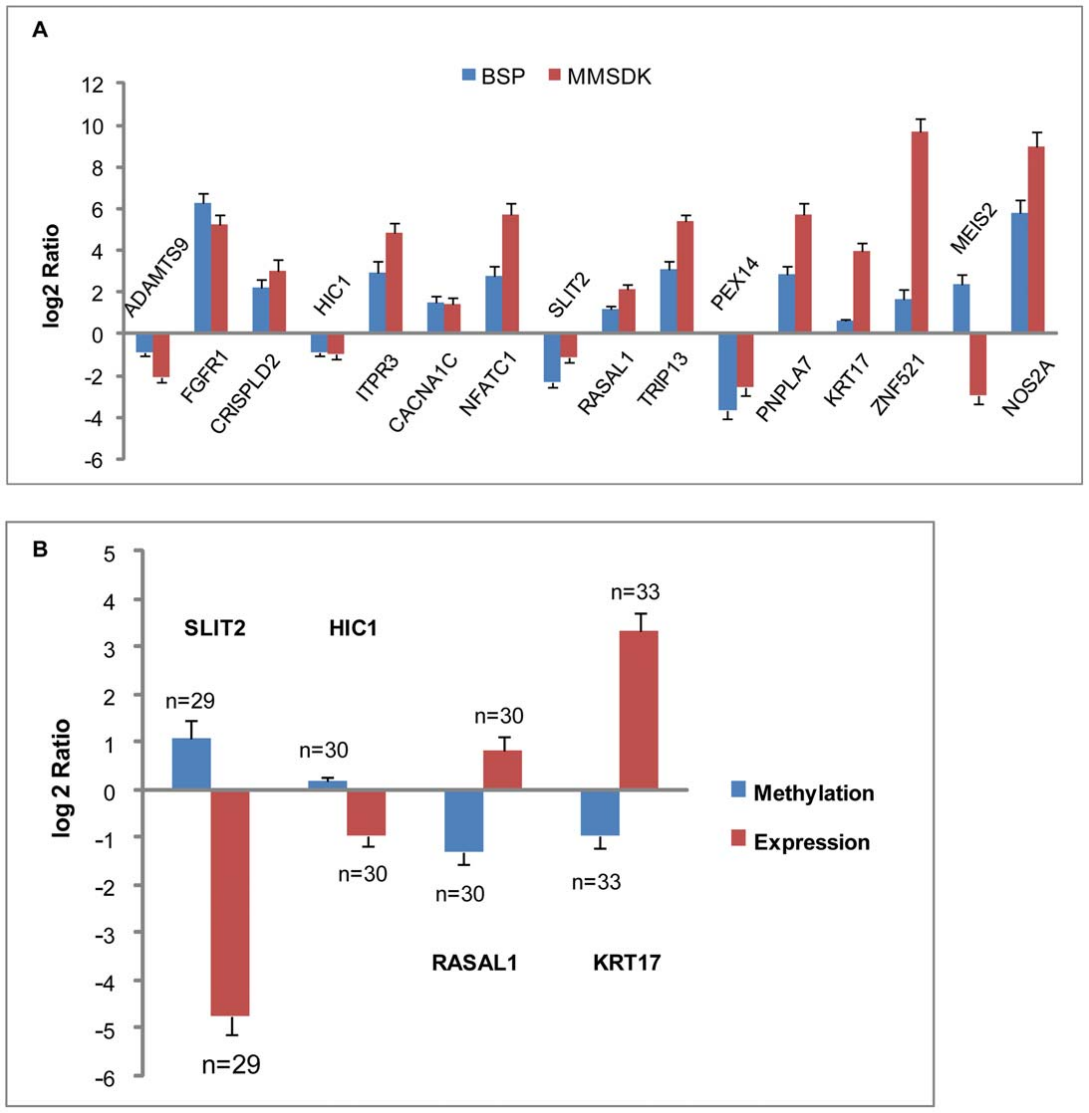
Validation results of BSP for DNA methylation and RT-qPCR for gene expression. A. Validation results of MMSDK with BSP. To compare the BSP-Sanger sequencing data and deep sequencing MMSDK data, the *MluI* loci that were determined to be differentially methylated on average by deep sequencing were validated using BSP. The height of the columns represents the log2-transformed average fold change (tumor/normal) in methylation level across the 9 patients. B. BSP and RT-qPCR results for four cancer-associated genes examined in 33 bladder cancer patients. The methylation levels of promoters of four selected genes (SLIT2, HIC1, RASRAl1, KRT17) and their expression level were evaluated in a panel of 33 samples. The height of the columns represents the log2 average fold change (tumor/normal) in methylation level (blue) or expression level (red) across all patients. The bars represent the standard error. The number of samples (n) used in the validation assay is indicated beside each standard error bar.

To further study the DNA methylation status at certain genomic regions, the genomic loci that correspond to the *MluI* recognition sites were associated with genes according to their relative position in the human genome. Up to 9034 individual genomic loci of 5656 unique genes were identified, based on the UCSC Genome Browser. We also identified 299 individual loci that correspond to 274 genes that show a significantly different DNA methylation level between tumors and controls (|log2Ratio|≥1, FDR P-value≤0.05) (Table S1). Of the significantly different loci, 36 loci were hypermethylated and 263 loci were hypomethylated in the tumor tissue samples compared with the matched normal adjacent tissue samples. BSP validation confirmed 16 randomly selected *MluI* genomic loci that were characterized as having different methylation levels with an average success rate of 93.8% (Figure 2A).

Between bladder cancer tissues and the matched normal controls, 2975 coding genes were detected as differentially expressed including 1560 up-regulated genes and 1415 down-regulated genes, and 196 miRNAs were differentially expressed including 156 up-regulated and 40 down-regulated miRNAs (|log2Ratio|≥1, FDR P-value≤0.05) (Table S2 and Table S3, a part of the mRNA and miRNA data came from our previous studies on mRNA [21] and miRNA [22] of bladder cancer). According to target prediction, 56 out of 196 differentially expressed miRNAs were predicted to target 285 different mRNAs (Table S4). As shown in Table 1, several solute carrier family members (SLC) were consistently under-expressed in the majority of bladder cancer tumors examined, including a facilitated glucose transporter (SLC2A4), a mitochondrial carrier (SLC25A25), a sodium/calcium exchanger (SLC8A1), a zinc transporter (SLC39A14), a cationic amino acid transporter (SLC7A2), a large neutral amino acid transporter (SLC43A1), and a monocarboxylic acid transporter (SLC16A1). In addition, we noted that two sodium-selective non-voltage-gated epithelial sodium channel (ENaC) genes (SCNN1B and SCNN1G) involved in regulating intracellular sodium homeostasis were significantly up-regulated in our study [23], [24]. A hepatocyte growth factor receptor (HGFR), also known as MET, was also sharply up-regulated in all of the tumors examined in our study as well. Consistent with recently published studies, miR-182, miR-183, miR-10a, miR-203, and miR-224 were all significantly up-regulated in the tumors, whereas miR-1, miR-143, miR-145 were significantly down-regulated in the tumor tissues [25]. Additionally, a typical down-regulation of miR-133a, miR-133b and miR-125b was detected in our study also observed formerly [26]. Remarkably, the tumor suppressors miR-1 and miR-133a were both significantly under-expressed in our study, resulting in the activation of the predicted target gene transgelin 2 (TAGLN2), which has been identified as a potential oncogene. This result is in agreement with existing knowledge about gene expression changes in bladder cancer [16].

**Table 1:** A collection of deregulated mRNAs and miRNAs detected by deep sequencing in bladder cancer.

mRNA	log2Ratio(Ca/N)	P-value	FDR	miRNA	log2Ratio(Ca/N)	P-value	FDR
SLC2A4	−2.76453644	4.97E-17	1.20E-15	miR-182	3.809715846	1.20E-11	4.64E-11
SLC8A1	−2.3659033	4.00E-21	1.09E-19	miR-183	3.357301827	3.95E-12	1.67E-11
SLC25A25	−2.02937463	1.82E-11	2.47E-10	miR-10a	2.638410238	0	0
SLC39A14	−1.88418005	3.07E-14	6.29E-13	miR-203	2.442035548	2.04E-12	9.06E-12
SLC7A2	−1.59152375	4.52E-05	0.00025956	miR-224	2.463582232	8.34E-14	4.65E-13
SLC43A1	−1.56691772	0.00307625	0.011617334	miR-1	−3.372834	0	0
SLC16A1	−1.49139112	1.31E-08	1.30E-07	miR-143	−2.3600868	0	0
SCNN1B	1.914656487	0	0	miR-145	−2.23501045	0	0
SCNN1G	3.05167703	7.72E-06	5.19E-05	miR-133a	−3.1155037	1.65E-38	1.35E-37
MET	4.242192911	0	0	miR-133b	−3.668212	1.67E-06	4.81E-06
TAGLN2	1.11741682	3.73E-13	6.12E-12	miR-125b	−2.32239525	0	0

Ca: bladder urothelial carcinoma, N: matched normal urothelium.

We performed integrated analysis of selected genes between DNA methylation and gene expression. In total, 4772 genes with both DNA methylation data and gene expression data were identified. As shown in supplementary Table S5, we identified 82 simultaneously differentially methylated and expressed genes by meeting the following conditions: |log2Ratio|≥1, adjusted P-value≤0.05, for both the DNA methylation and gene expression data. Several well-known cancer-associated genes (including ADAMTS9, CCND1, HIC1, etc.) exhibited consistently disrupted methylation and expression statuses in the majority of the bladder urothelium cancer samples.

### Gene ontology (GO) and KEGG pathway enrichment analysis

To generate further insight view of pathway perturbation underlying bladder cancer, we performed GO and KEGG pathway analysis on gene sets containing differentially methylated sites, differentially expressed genes identified from mRNA sequencing and the target genes of deregulated miRNAs.

Among all the genes containing divergent DNA methylation loci, the GO analysis for “biological processes” and “molecular function” showed a significant enrichment of 19 and 13 GO terms, respectively, using a FDR P-value cutoff of 0.05. The most significant enriched GO terms in “biological process” were “neuron development”, suggesting a critical role for differential methylation in neurogenesis. Similarly, only the “axon guidance” pathway was significantly enriched (FDR P-value: 3.29E-13) via KEGG pathway analysis, reflecting a concordance with GO analysis result. This result suggests an important mechanism of epigenetic abnormality in neuron genes that play a role in bladder cancer, as cancer-related axonogenesis and neurogenesis has been observed in prostate cancer [27]. The remaining enriched GO terms from the “molecular function” analysis were primarily related to various transporter activities (Table S6).

For differentially expressed genes, 226 GO terms in the category of “biological process” and 8 GO terms in the category of “molecular function” were significantly enriched at a FDR threshold of P-value≤0.05 (Table S7). From the “biological processes” analysis, a prevalence of negative regulation genes was observed in the typical down-regulated genes. Meanwhile, we also noticed a significant frequent occurrence of genes involved in the regulation of the cell cycle and mitosis among the up-regulated genes. The enriched GO terms of “molecular function” were primarily related to “protein kinase activity”. KEGG pathway analysis also revealed that several well-known cancer-associated pathways were significantly disturbed (FDR P-value≤0.05), e.g. ECM-receptor interaction, cell cycle, p53 signaling, and Calcium signaling pathway. Various metabolic pathways (such as those involved in amino acid metabolism) were also observed to be enriched in the deregulated genes identified by our study (FDR P-value≤0.05).

For the target genes of all the deregulated miRNAs, 149 GO terms were enriched from the “biological processes” analysis using the similar threshold. Generally, cellular process, biological and metabolic process terms were found to be enriched among the miRNA-target genes that were deregulated in our study. The “molecular function” analysis revealed 11 GO terms that were significantly enriched by a FDR threshold of P-value≤0.05. The most significantly enriched GO term was “protein kinase activity”, suggesting a role in kinase activity regulation. In addition, pathway analysis of the targets of the deregulated miRNAs also showed a significant perturbation of cell cycle, axon guidance and MAPK signaling pathways (adjusted P-value≤0.05) (Table S8).

To further scrutinize the connection among the three categories of -omics, the GO terms of molecular functions and biological processes, as well as the KEGG pathways that were enriched in more than one category were further analyzed. The GO and KO terms enriched in more than one category are presented in Table S9. As many as 106 enriched GO terms and KEGG pathways were enriched in our study, the majority of which were tightly associated with cancer. Remarkably, overlapping of significantly enriched GO terms and KEGG pathways among three categories of -omics was carried out subsequently, including a key neural development pathway named “axon guidance”. Furthermore, the 7 enriched “biological process” GO terms and the single “molecular function” GO term were primarily related to “neurogenesis” and “cell differentiation”, suggesting a combined regulation of key processes and functions by differential methylation and mRNAs and miRNAs expression regulation.

### Cancer-related genes significantly deregulated in bladder cancer

Through the GO term enrichment and KEGG pathway analysis of selected differentially regulated genes, we observed that certain genes were both differentially methylated and expressed which were involved in significant perturbation pathways, and these pathways were closely connected to various tumorigenesis processes. For instance, SLIT2 was involved in Axon guidance pathway, and CCND1 was involved in cell cycle and p53 signaling pathway. Besides, we identified several canonical cancer-associated genes that were not enriched in the significantly disturbed pathways, such as ADAMTS9, HIC1, RASAL1, in both the methylation statuses and expression data (Table 2).

**Table 2:** A collection of significantly deregulated genes detected by deep sequencing in bladder cancer.

Gene	Methylation			Expression		
	log2 Ratio(Ca/N)	P-value	FDR	log2 Ratio(Ca/N)	P-Value	FDR
HIC1	−1.43	3.2E-13	4.94E-11	−1.84	3.98E-05	0.0002313
SLIT2	−1.14	9.46E-09	6.15E-07	−2.90	9.68E-08	8.54E-07
RASAL1	1.11	1.99E-07	1.02E-05	3.69	5.31E-04	2.42E-03
KRT17	1.08	1.36E-04	3.32E-03	2.19	2.49E-12	3.66E-11
ADAMTS9	−1.52	0	0	−1.46	1.70E-03	6.85E-03
CCND1	1.51	3.53E-57	2.66E-54	1.84	0	0
ITPR3	3.71	2.75E-12	3.40E-10	1.32	1.11E-06	8.44E-06
PEX14	−1.50	2.41E-06	9.30E-05	−1.51	1.85E-10	2.27E-09
MEIS2	−1.40	5.78E-04	1.12E-02	−1.64	6.80E-08	6.08E-07

Ca: bladder urothelial carcinoma, N: matched normal urothelium.

As DNA methylation aberration in gene promoters is reported to influence their expression levels, those methylation sites located in promoter regions were analyzed in details. Hypermethylated in cancer 1 (HIC1) and slit homolog 2 (SLIT2) were identified as having differentially up-regulated methylation status of their promoter regions and dramatically down-regulated expression level across all the bladder urothelial carcinoma tissue samples. Besides, we found that RAS protein activator like 1 (RASAL1) and keratin 17 (KRT17) were clearly hypomethylated in the promoter region and over-expressed in the tumor samples compared to the controls. These four genes have been identified as potential tumor suppressor genes or oncogenes in studies of other tumor types [28], [29], [30], [31], but have not been implicated in bladder cancer.

To further investigate the methylation and expression alterations of HIC1, SLIT2, RASAL1, and KRT17, massive bisulfite sequencing PCR and reverse transcription qPCR were performed in matched pairs of cancer and normal adjacent tissues from additional ∼33 bladder urothelial carcinoma patients (see methods for details). As shown in Figure 2B, aberration of regional promoter methylation was tightly associated with altered gene expression in these four genes in bladder cancer. In the human bladder urothelial carcinoma samples examined, increased HIC1 promoter methylation was detected in 100% (30/30) of the samples and reduced expression was observed in 70.0% of the 30 tumor samples. Hypermethylation of the SLIT2 promoter was observed in 58.6% (17/29) cases, and a significant reduction of expression was detected at a rate of 86.2% (25/29). The decreased methylation frequency for RASAL1 was 96.7% (29/30), and KRT17 had a frequency of 93.9% (31/33). The corresponding increased gene expression rates for RASAL1 and KRT17 were 63.3% (19/30) and 78.8% (26/33), respectively. In total, increased HIC1 and SLIT2 promoter methylation and decreased expression were observed in 70.0% and 51.7% of the tumor samples, respectively. Meanwhile, hypomethylation of the RASAL1 and KRT17 promoters and down-regulation of their expression were detected at rates of 63.3% and 75.8%, respectively.

## Discussion

MMSDK technology provides an efficient and cost-effective method to analyze genome-wide DNA methylation profiles with a relatively high sensitivity [32]. The methylation level detected by MMSDK correlates well with those validation results of bisulfite sequencing PCR in randomly selected *MluI* loci in our study. DGE sequencing is also a useful and sensitive tool that allows for accurate detection of a wide range of expression levels of both mRNAs and miRNAs and thus allows for differential expression analysis of a greater range of transcripts [33] [34]. These two technologies both couple tag generation via enzyme digestion with next-generation deep sequencing and allow for a sensitive comparative analysis of DNA methylation and gene expression.

In general, a similar genome-wide methylation pattern was found between bladder tumor and normal adjacent tissues. Methylation levels at CpG rich and poor promoters, exons, and introns were also similar between tumor and normal tissues, supporting the above findings regarding the similar global methylation patterns. In gene level, it's well known that inactivation of tumor-suppressor genes by site-specific hypermethylation contributes to the initiation and progression of human malignancies [2], [35]. Hypermethylation of HIC1 and the associated decrease in HIC1 expression is a common feature in several cancers, such as human breast cancer [28], acute myeloid leukemia [36], and prostate cancer [37]. In bladder cancer, a similar study has also been carried out that quantitative methylation-specific PCR was done at 17 gene promoters including HIC1 in 96 malignant and 30 normal urothelial samples [38]. found that the methylation frequencies of HIC1 promoter in malignant and normal urothelium were 21.9% and 16.7%, respectively. They didn't show significant difference between malignant and normal urothelial tissue. Here, we reported the hypermethylation of HIC1 and the resulting decreased expression of HIC1 mRNA in bladder cancer. The difference between our study and Yates DR's study might be explained by the different resolution of technologies, as we applied high-resolution deep sequencing technology while Yates DR's group applied relatively low-solution technology of quantitative MSP, or by different sampling strategy, as we collected exactly matched pair samples of tumor tissues and normal adjacent tissues from the same patients, while the most of tumor tissues that Yates DR's group collected didn't have matched normal tissues. HIC1 is a candidate tumor suppressor located at 17p13.3, a region frequently found to be hypermethylated or deleted in many types of prevalent human tumors [39]. The entire HIC1 gene region is encompassed within a dense CpG island that is normally unmethylated. HIC1 (hypermethylated in cancer 1) has been demonstrated to be involved in a complex signaling pathway that impairs p53-mediated apoptotic DNA damage response in the process of tumorigenesis [40], [41]. Epigenetic inactivation of HIC1 would induce the deacetylation of p53, disrupting its function and resulting in a reduced apoptotic response to DNA damage, leading to oncogenesis and promoting tumor progression [35], [41], [42]. Therefore, our research indicated that promoter CpG island hypermethylation caused HIC1 transcription inactivation and might disrupt the complex HIC1-p53 signaling pathway in bladder cancer.

The Slit gene family (SLIT1, SLIT2, and SLIT3) is a recently characterized family of secreted repellents in axon guidance and neuronal migration during the development of the central nervous system [43], [44]. A number of studies have demonstrated that SLIT2 is epigenetically silenced by hypermethylation of the promoter region in a broad spectrum of other tumors, such as lung, breast, glioma, colon, and cervical cancers, leading to inactivation of SLIT2 expression [29], [45], [46], [47]. The conditioned medium from SLIT2-transfected cells reduces cell growth and induces apoptosis of colorectal carcinoma cell lines, implicating that SLIT2 has tumor-suppressor activities [45]. Additionally, several studies have demonstrated that SLIT2 inhibits tumor growth and metastasis by a mechanism that involves the regulation of tumor migration, invasion, apoptosis, and growth in breast cancer, fibrosarcoma, and squamous Cell Carcinoma [48], [49]. Hypermethylation at the promoters of genes (SLIT1, SLIT2, SLIT3, ROBO1, and ROBO3) involved in Slit-Robo pathway resulting in down-regulated gene expression was also indentified in invasive cervical cancer [46]. This work identified that the tumor suppressor gene, SLIT2, which inhibited tumor growth and metastasis, was found inactivated by promoter hypermethylation in bladder urothelial carcinoma as well.

Previous studies identified hypermethyled promoter regions in some commonly methylated genes (RASSF1A, p16, MLH1, MGMT, HOXA9) in different types of cancers. We tried to confirm such results in bladder urothelial carcinoma as well. Unfortunately, there were no *MluI* enzyme digestion sites to be found in the promoters of these genes, thus we couldn't appropriately address the methylation levels of promoters in these commonly methylated genes due to technological limitations. However, we found that MLH1 and HOXA9, but not the others of these commonly methylated genes, were significantly under-expressed in bladder cancer, which were in agreement with previous studies [10], [50].

In contrast to hypermethylation, DNA hypomethylation contributes to tumorigenesis by activating proto-oncogenes [5], which contributes to cancer development and progression [51]. As was mentioned in the results of massive BSP and RT-qPCR tests, RASAL1 methylation levels were significantly down-regulated, and the expression levels was dramatically increased in bladder cancer cells in 66.7% and 53.3%, respectively, of the patients with bladder cancer examined. However, conflicting results indicating that RASAL1 was silenced through promoter CpG methylation in multiple tumors [52] and that a reduction in RASAL1 expression might contribute to carcinogenesis and was associated with tumor progression [30], [53] have been published. RASAL1 is a RAS-GTPase-activating protein that switches off RAS activity by converting the GTPase from its active, GTP-bound form to its inactive, GDP-bound form [54]. The RAS gene family is well known as one of the most frequently activated oncogenes in human cancers [55]. Surprisingly, RASAL1, the RAS signal terminator, was highly over-expressed and hypomethylation of the promoter was also observed in the majority of the examined bladder cancer tissues in our research. Further studies are needed to address the mechanisms that underlie the RASAL1 over-expression and epigenetic aberration in bladder cancer.

Keratin 17, also known as cytokeratin 17, is involved in the formation and maintenance of various skin appendages, especially in determining the shape and orientation of hair. The expression of KRT17 has been found to be up-regulated in cancerous tissues in cervical [56], tongue [57], oral [58], and esophageal squamous cell carcinomas [59]. Increased expression of keratin 17 in endothelial cells may contribute to angiogenesis [60] and may promote epithelial proliferation and tumor growth [31]. Keratin 17 has also been reported as a potential diagnostic marker for oral squamous cell carcinoma [58], and it might be linked to the clinical progression and differentiation of cervical carcinoma [61], [62]. However, there are few reports on the epigenetic regulation and expression of keratin 17 in bladder cancer. In this study, we identified an increase of KRT17 expression in bladder cancer tissues compared with their normal counterparts. We also observed hypomethylation of the KRT17 promoter in bladder urothelial carcinoma. KRT17 might be a putative oncogene that is activated through hypomethylation at the promoter.

Our study also highlights the importance of investigating the functional roles of mRNAs and miRNAs in the tumorigenesis of bladder urothelial carcinoma. Met proto-oncogene (MET), a hepatocyte growth factor receptor (HGFR) that has been suggested to play an important role in the development of various epithelial and nonepithelial tumors, was found to be significantly over-expressed in our study of bladder cancer, consistent with previous reports on bladder cancers [63], haematopoietic malignancies [64], and renal cell carcinomas [65]. MET is constitutively activated as a proto-oncogenic receptor tyrosine kinase, which promotes invasion and migration by activating a variety of signal transduction pathways in numerous types of carcinomas [66]. Interestingly, the down-regulation of miR-1, which targets MET, was observed in our study, similar to previous studies in lung cancer and human Rhabdomyosarcoma [67], [68]. MiR-1 is thought to inhibit cell proliferation and migration by negatively regulating the c-Met signaling pathway. Therefore, characterization of the oncogenes targeted by miRNAs would be of great value in improving our understanding of the pathogenic mechanisms underlying bladder cancer.

It is widely acknowledged that disrupted pathways, not just deregulated individual genes, drive the tumorigenesis process. In our search for functional relevance of altered methylation levels and gene expression, we found that KEGG “axon guidance” pathway was significantly enriched in the differentially expressed genes (mRNAs and miRNAs) and that there were overlaps with the pathways enriched for relevant genes with differentially methylated loci. Axon guidance represents a key stage during which axons extend to their correct targets during the formation of neuronal networks and related molecules were thought to be widely expressed and involved in tumor development, angiogenesis and metastasis [69]. We also observed such overlaps for significantly enriched GO terms, which primarily related to “neurogenesis” and “cell differentiation” (e.g. neuron differentiation, neurogenesis, positive regulation of cell differentiation) among three types of omics data. Different categories of omics might co-regulate cellular processes simultaneously via overlapping pathways, suggesting that a complex means of multilevel regulation is involved in tumor progression.

In summary, we systematically profiled the DNA methylation and expression patterns of both miRNAs and mRNAs on a genome-wide scale in bladder urothelial carcinoma using deep sequencing technology. We characterized the profiles between methylome and transcriptome in bladder urothelial carcinoma, identified a set of significantly enriched key pathways, and screened four simultaneously aberrantly methylated and expressed genes. It is noted that the number of bladder cancer patients in our study was relatively small. This observation requires further confirmation in a larger panel of tumors. Our results provide a new perspective for the application of integrated analysis of multi-omics to identify complex multi-level regulation means involved in cancer.

## Materials and Methods

### Clinical sample collection

The tumor tissues and matched normal adjacent tissues from the patients with transitional cell carcinoma of the bladder in this study were obtained from the biobank (CXC201005260001A) of Urinogenital Cancer Genomics Consortium (UCGC) in China. Detailed information of the 42 patients (9 for MMSDK and DGE sequencing and 33 for validation screen) is summarized in Table S10. Specimens were deposited in RNALater (Qiagen, Germany) or snap-frozen in liquid nitrogen and subsequently stored at −80°C. Hematoxylin-eosin (HE)-stained sections were examined for tumor cell percentage, and tumor tissues containing more than 85% tumor cells were selected for further study. The matched normal adjacent tissues were defined as bladder transitional epithelial tissues located at least 2 centimeters outside of visible bladder cancer lesions. The collection and use of the samples were reviewed and approved by Institutional Ethics Committees of Peking University Shenzhen Hospital (Shenzhen, China), and written informed consent from all the patients was obtained.

### DNA and RNA extraction

Genomic DNA from bladder urothelial carcinomas and normal adjacent tissues were prepared using DNeasy Blood & Tissue Kit (Qiagen, Germany) according to the manufacture's protocol, DNA was then dissolved in DNase-free water and the concentrations were determined before being stored at −80°C. Total RNA was extracted from bladder cancer and normal adjacent tissues using TRIZOL (Invitrogen, US) according to the manufacture's protocol and evaluated using Agilent 2100 Bioanalyzer (Agilent Technologies, US).

### MMSDK and statistical analysis

MMSDK combines the use of methylation-sensitive restriction enzyme digestion and the second generation sequencing technique (Illumina Genome Analyzer IIx) [32]. Briefly, 3 µg of genomic DNA was digested with *MluI* (recognition sequence: ACGCGT) (NEB, US). The *MluI*-digested genomic DNA was ligated to biotinylated linkers and fragmented by *NlaIII* cleavage (NEB, US). DNA fragments were captured with streptavidin-conjugated magnetic beads and then bound to the first adapter, which contains a *MmeI* restriction enzyme recognition site. Bounded DNA was then digested with *MmeI* (NEB, US), which generated a 17–18 nt library, followed by ligating to the P7 adapter. DNA fragments were amplified by PCR for 15 cycles using Phusion DNA polymerase (Finnzymes, Finland) with universal primer and index primers. Gel electrophoresis was used to isolate a 130–150 bp gel slice, and the isolated fragment was gel-purified using Spin-X Centrifuge Tube Filters (Corning, US). Each MMSDK library was examined by Agilent 2100 Bioanalyzer (Agilent, US) and real-time PCR for quality control, after which cluster generation and a standard sequencing were performed on the Illumina Cluster Station and Genome Analyzer IIx (Illumina, US) following manufacturer's protocol.

We firstly filtered the data to generate effective reads by removing the low-quality sequences, as well as 3′ adaptor sequence, leaving tags of 17–18 nt. A virtual *MluI* and *NlaIII* digested DNA fragment library was constructed as a reference genome. Digital simulated analysis showed 21038 *MluI* recognition sites in the human genome sequence (reference build hg18). A total of 42126 virtual fragments were obtained from simulated digestion with *MluI* and *NlaIII* under the hypothesis that all cytosines are unmethylated. Only 694 *MluI* fragments lacked an internal *NlaIII* recognition site. We found 2942 genomic loci to be located in CGIs defined by the following three criteria: GC content ≥50%, ratio of the observed CpGs to the expected CpGs>0.6, length>200 bp. The corresponding CpG Islands annotation file was downloaded from the UCSC Table Browsers [70]. MAQ (Mapping and Assembly with Qualities) was used to map all tags to this library while 1 bp mismatch was allowed [71]. We extracted high-confidence mapped tags with a mapping quality score greater than 20 to ensure unambiguous mapping and then averaged the mapped tags across the 9 tissues at each *MluI* recognition site. In our experiment, the tags represent unmethylated sites. The corresponding gene annotation of those genomic loci was simultaneously found using the UCSC Genome Browser (http://genome.ucsc.edu/). The averaged tag numbers were normalized (divide high-quality tags by total tags and multiplied by one million) and were used to compared the methylation level between tumors and normal adjacent tissues. The Poisson distribution was used to calculate the P-value for each *MluI* recognition site in the genome to detect differentially methylation level between bladder cancer tissues and matched normal adjacent tissues [72]. The False Discovery Rate (FDR) adjustment was performed to judge the significance of difference in methylation level in multiple test [73]. Genomic loci with a calculated FDR≤0.05 and a |log_2_Ratio|≥1 were defined as being differentially methylated.

### Digital gene expression (DGE) sequencing of mRNA and the resulting statistical analysis

To detect the expression of mRNA in the tumor tissues and matched normal adjacent tissues, 4 µg of the total RNA isolated from each sample was used for DGE sequencing. Briefly, after synthesis of double-stranded cDNA using oligo(dT)_18_ beads (Invitrogen, US), the cDNA was digested with *NlaIII* and ligated to the first adapter (GEX adapter 1) that contained a restriction site for *MmeI*. After dephosphorylation with the alkaline phosphatase, CIAP (Invitrogen, US), the purified MmeI-digested products were linked to the second adaptor (GEX index adapter) containing degenerate 2 bp 3′ overhangs. The double adapter-flanked tags from the mRNAs were then amplified by PCR using Phusion DNA polymerase (Finnzymes, Finland) and GEX PCR primers following the manufacturer's protocol. The resulting ∼85-bp PCR products were ethanol precipitated and purified by gel electrophoresis and Spin-X filter columns (Corning, US). Finally, the mRNA libraries were sequenced on the Illumina Cluster Station and Genome Analyzer IIx (Illumina, US) following the manufacturer's protocol.

Prior to the statistical analysis, potentially erroneous tags (single copy tags and tags consisting of adapter sequences or containing unknown sequences, ‘N’) were filtered out. All of the 17 bp sequences next to the potential *NlaIII* restriction sites in the human reference genome (hg18) and the CATG restriction enzyme-digested site were extracted and concatenated to form a new reference sequence [74]. Tags were mapped to the constructed reference sequence using SOAP V2.0 [75] allowing no more than 1 bp mismatch. Only uniquely mapped tags were used for the gene expression analysis. As with the MMSDK statistical analysis, all of the mapped tags were averaged among the 9 samples. Normalized TPM (high-quality tags per million tags) values were used to compare the gene expression levels between tumors and normal adjacent tissues. The expression fold change (tumor versus normal) for each gene was calculated as the log_2_Ratio using the TPM values. We subsequently performed Poisson distribution test to determine the differentially expressed genes [72]. The resulting P-values for all genes were corrected for multiple testing using a FDR (false discovery rate) adjustment [73]. The mRNAs with a calculated FDR≤0.05 and a |log_2_Ratio|≥1 were defined as differentially expressed mRNAs.

### DGE sequencing of miRNA and statistical analysis

For miRNA sequencing, 5 µg of total RNA from each sample was ligated to both a 5′ adapter and 3′ adapter for reverse transcription using Superscript II (Invitrogen, US). Subsequently, the reverse transcripted products were amplified by PCR and evaluated by gel electrophoresis. After obtaining a ∼92 bp DNA band from a 6% PAGE gel, the PCR products were ethanol precipitated and purified using Spin-X filter columns (Corning, US). Finally, miRNA libraries were performed to cluster generation and standard cycle of sequencing on the Illumina Cluster Station and Genome Analyzer IIx following manufacturer's protocol.

Low quality reads were trimmed and the adapter sequences were accurately clipped using a dynamic programming algorithm prior to subsequent statistical analysis. After elimination of the duplicate reads, the remaining reads of at least 18 nt were mapped to a human reference genome (hg18) using SOAP V2.0. We removed tags originating from protein-coding genes, repeat sequences, rRNA, tRNA, snRNA, and snoRNA by mapping the short read tags to UCSC RefGene, RepeatMasker and NCBI Refseq, as well as our in-house ncRNA annotation datasets compiled from the NCBI GenBank database (http://www.ncbi.nih.gov). The same pipeline used for the DGE mRNA differential expression analysis was also used for the miRNA expression analysis.

To identified the target genes of deregulated miRNAs, we firstly compared the predictions with DIANA-microT v3.0 [76], TargetScan 5.1 [77], and PicTar [78]. These tools are characterized by their high precision [79], and the intersecting results of two of these three tools were obtained, To reduce the false positive rate, we generated the union set of two experimentally supported database, TarBase V5.0 [80] and miRecords V3 [81]. The intersection of the results from these two steps was selected as representing the robust target genes and was used for further analysis.

### GO and KEGG pathway analysis

GO and KEGG pathway analysis was performed by Cytoscape V2.7 (http://cytoscape.org/) with the ClueGo V1.3 plug-in [82]. ClueGO determines the distribution of the target gene list across the GO terms and pathways. The P-value was calculated using a right-side hypergeometric tests, and Benjamini-Hochberg adjustment was used to multiple test correction. An adjust P-value≤0.05 indicates a statistically significant deviation from the expected distribution, and thus corresponding GO terms and pathways were enriched in target genes. We analyzed all of the differentially methylated loci, expressed genes, and target genes of deregulated miRNAs using GO and KEGG pathway analysis.

### Validation of DNA methylation status and the expression of mRNAs by BSP and RT-qPCR

To validate the methylation status of genes detected as being differentially methylated by deep sequencing, we combined BSP and clone sequencing for 16 differentially methylated genes in both tumor and normal adjacent tissue samples from the 9 bladder cancer patients. In brief, 1 µg of genome DNA from each sample was bisulfite converted according to the manufacturer's protocol (Qiagen, Germany). Primers for BSP were designed by MethPrimer (Table S11) (http://www.urogene.org/methprimer/index1.html). The bisulfite conversion products were amplified using the following PCR program: 94°C for 4 min, followed by 35 cycles of 94°C for 30 sec, 55°C for 30 sec, 72°C for 30 sec and then extension at 72°C for 10 min. Then, bisulfite PCR products were cloned into a T vector (TaKaRa, Japan) after purification, and 30 clones were selected to further Sanger sequenced for at each validated gene.

BSP and RT-qPCR were used to further evaluate the methylation and expression status of the HIC1, SLIT2, RASAL1, and KRT17 genes in an additional 33 bladder cancer patients. The BSP products were cloned into a T vector and 20 clones were further Sanger sequenced for each gene. The BSP primers and programs used were the same as described previously. For RT-qPCR, 1 µg of total RNA from each sample was reverse transcribed to cDNA using a reverse transcription kit according to the manufacturer's protocol (Promega, US). The reverse transcription products were amplified using the following PCR program: 50°C for 2 min,94°C for 2 min, followed by 40 cycles of 94°C for 30 sec, 55°C for 20 sec, and 72°C for 15 sec. All of the primers used in the validation assays are listed in Table S11.

## Supporting Information

Table S1 **Differentially methylated genomic loci located in genic regions.**
(XLS)

Table S2 **mRNAs that are differentially expressed in bladder cancer.**
(XLS)

Table S3 **miRNAs that are differentially expressed in bladder cancer.**
(XLS)

Table S4 **Differently expressed miRNAs and miRNA targets in bladder cancer.**
(XLS)

Table S5 **Differentially methylated and expressed genes in bladder cancer.**
(XLS)

Table S6 **Enriched GO terms and KEGG pathways in differentially methylated genomic loci in bladder cancer.**
(XLS)

Table S7 **Enriched GO terms and KEGG pathways in differentially expressed genes in bladder cancer.**
(XLS)

Table S8 **Enriched GO terms and KEGG pathways in targets of differentially expressed miRNAs in bladder cancer.**
(XLS)

Table S9 **GO and KO terms enriched in multiple data types (methylation, mRNAs, and miRNAs) in bladder cancer.**
(XLS)

Table S10 **Clinical information on the 42 patients sequenced or validated in this study.**
(DOC)

Table S11 **BSP and RT-qPCR primers used in the validation assays.**
(DOC)

## References

[1] Shen L, Toyota M, Kondo Y, Lin E, Zhang L (2007). Integrated genetic and epigenetic analysis identifies three different subclasses of colon cancer.. Proc Natl Acad Sci U S A.

[2] Sugimura T, Ushijima T (2000). Genetic and epigenetic alterations in carcinogenesis.. Mutat Res.

[3] Jones PA, Baylin SB (2002). The fundamental role of epigenetic events in cancer.. Nat Rev Genet.

[4] Eden A, Gaudet F, Waghmare A, Jaenisch R (2003). Chromosomal instability and tumors promoted by DNA hypomethylation.. Science.

[5] Clark SJ, Melki J (2002). DNA methylation and gene silencing in cancer: which is the guilty party?. Oncogene.

[6] Croce CM (2009). Causes and consequences of microRNA dysregulation in cancer.. Nat Rev Genet.

[7] Jemal A, Bray F, Center MM, Ferlay J, Ward E (2011). Global cancer statistics.. CA Cancer J Clin.

[8] Oosterlinck W, Lobel B, Jakse G, Malmstrom PU, Stockle M (2002). Guidelines on bladder cancer.. Eur Urol.

[9] Wolff EM, Chihara Y, Pan F, Weisenberger DJ, Siegmund KD (2010). Unique DNA methylation patterns distinguish noninvasive and invasive urothelial cancers and establish an epigenetic field defect in premalignant tissue.. Cancer Res.

[10] Reinert T, Modin C, Mansilla Castano F, Lamy P, Wojdacz TK (2011). Comprehensive genome methylation analysis in bladder cancer;identification and validation of novel methylated genes and application of these as urinary tumor markers.. Clin Cancer Res.

[11] Marsit CJ, Houseman EA, Schned AR, Karagas MR, Kelsey KT (2007). Promoter hypermethylation is associated with current smoking, age, gender and survival in bladder cancer.. Carcinogenesis.

[12] Jarmalaite S, Jankevicius F, Kurgonaite K (2005). Promoter Hypermethylation Is Associated With Tumor Location, Stage, and Subsequent Progression in Transitional Cell Carcinoma.. Journal of Clinical Oncology.

[13] Jarmalaite S, Jankevicius F, Suziedelis K, Mutanen P, Husgafvel-Pursiainen K (2008). Promoter Hypermethylation in Tumour Suppressor Genes Shows Association with Stage, Grade and Invasiveness of Bladder Cancer.. Oncology.

[14] Marsit CJ, Houseman EA, Christensen BC, Gagne L, Wrensch MR (2010). Identification of methylated genes associated with aggressive bladder cancer.. PLoS One.

[15] Uchida Y, Chiyomaru T, Enokida H, Kawakami K, Tatarano S (2011). MiR-133a induces apoptosis through direct regulation of GSTP1 in bladder cancer cell lines.. Urol Oncol.

[16] Yoshino H, Chiyomaru T, Enokida H, Kawakami K, Tatarano S (2011). The tumour-suppressive function of miR-1 and miR-133a targeting TAGLN2 in bladder cancer.. Br J Cancer.

[17] Ayala de la Pena F, Kanasaki K, Kanasaki M, Tangirala N, Maeda G (2011). Loss of p53 and acquisition of angiogenic microRNA profile is insufficient to facilitate progression of bladder urothelial carcinoma in situ to invasive carcinoma.. J Biol Chem.

[18] Wang G, Zhang H, He H, Tong W, Wang B (2010). Up-regulation of microRNA in bladder tumor tissue is not common.. Int Urol Nephrol.

[19] Gottardo F, Liu CG, Ferracin M, Calin GA, Fassan M (2007). Micro-RNA profiling in kidney and bladder cancers.. Urol Oncol.

[20] Dyrskjot L, Ostenfeld MS, Bramsen JB, Silahtaroglu AN, Lamy P (2009). Genomic Profiling of MicroRNAs in Bladder Cancer: miR-129 Is Associated with Poor Outcome and Promotes Cell Death In vitro.. Cancer Research.

[21] Li X, Chen J, Hu X, Huang Y, Li Z (2011). Comparative mRNA and microRNA Expression Profiling of Three Genitourinary Cancers Reveals Common Hallmarks and Cancer-Specific Molecular Events.. PLoS One.

[22] Han Y, Chen J, Zhao X, Liang C, Wang Y (2011). MicroRNA expression signatures of bladder cancer revealed by deep sequencing.. PLoS One.

[23] Yamamura H, Ugawa S, Ueda T, Shimada S (2008). Expression analysis of the epithelial Na+ channel delta subunit in human melanoma G-361 cells.. Biochem Biophys Res Commun.

[24] Frateschi S, Charles R-P, Hummler E (2010). The Epithelial Sodium Channel ENaC and its Regulators in the Epidermal Permeability Barrier Function.. The Open Dermatology Journal.

[25] Friedman JM, Liang G, Liu CC, Wolff EM, Tsai YC (2009). The putative tumor suppressor microRNA-101 modulates the cancer epigenome by repressing the polycomb group protein EZH2.. Cancer Res.

[26] Ichimi T, Enokida H, Okuno Y, Kunimoto R, Chiyomaru T (2009). Identification of novel microRNA targets based on microRNA signatures in bladder cancer.. Int J Cancer.

[27] Ayala GE, Dai H, Powell M, Li R, Ding Y (2008). Cancer-related axonogenesis and neurogenesis in prostate cancer.. Clin Cancer Res.

[28] Fujii H, Biel MA, Zhou W, Weitzman SA, Baylin SB (1998). Methylation of the HIC-1 candidate tumor suppressor gene in human breast cancer.. Oncogene.

[29] Dallol A, Da Silva NF, Viacava P, Minna JD, Bieche I (2002). SLIT2, a human homologue of the Drosophila Slit2 gene, has tumor suppressor activity and is frequently inactivated in lung and breast cancers.. Cancer Res.

[30] Ohta M, Seto M, Ijichi H, Miyabayashi K, Kudo Y (2009). Decreased Expression of the RAS-GTPase Activating Protein RASAL1 Is Associated With Colorectal Tumor Progression.. Gastroenterology.

[31] Depianto D, Kerns ML, Dlugosz AA, Coulombe PA (2010). Keratin 17 promotes epithelial proliferation and tumor growth by polarizing the immune response in skin.. Nat Genet.

[32] Li J, Gao F, Li N, Li S, Yin G (2009). An improved method for genome wide DNA methylation profiling correlated to transcription and genomic instability in two breast cancer cell lines.. BMC Genomics.

[33] Morrissy AS, Morin RD, Delaney A, Zeng T, McDonald H (2009). Next-generation tag sequencing for cancer gene expression profiling.. Genome Res.

[34] Cullum R, Alder O, Hoodless PA (2011). The next generation: Using new sequencing technologies to analyse gene regulation.. Respirology.

[35] Baylin SB, Ohm JE (2006). Epigenetic gene silencing in cancer - a mechanism for early oncogenic pathway addiction?. Nat Rev Cancer.

[36] Melki JR, Vincent PC, Clark SJ (1999). Cancer-specific region of hypermethylation identified within the HIC1 putative tumour suppressor gene in acute myeloid leukaemia.. Leukemia.

[37] Kekeeva TV, Popova OP, Shegai PV, Alekseev B, Adnreeva I (2007). [Abberant methylation of p16, HIC1, N33 and GSTP1 genes in tumor epitelium and tumor-associated stromal cells of prostate cancer].. Mol Biol (Mosk).

[38] Yates DR, Rehman I, Abbod MF, Meuth M, Cross SS (2007). Promoter hypermethylation identifies progression risk in bladder cancer.. Clin Cancer Res.

[39] Wales MM, Biel MA, el Deiry W, Nelkin BD, Issa JP (1995). p53 activates expression of HIC-1, a new candidate tumour suppressor gene on 17p13.3.. Nat Med.

[40] Chen W, Cooper TK, Zahnow CA, Overholtzer M, Zhao Z (2004). Epigenetic and genetic loss of Hic1 function accentuates the role of p53 in tumorigenesis.. Cancer Cell.

[41] Chen WY, Wang DH, Yen RC, Luo J, Gu W (2005). Tumor suppressor HIC1 directly regulates SIRT1 to modulate p53-dependent DNA-damage responses.. Cell.

[42] Fleuriel C, Touka M, Boulay G, Guerardel C, Rood BR (2009). HIC1 (Hypermethylated in Cancer 1) epigenetic silencing in tumors.. Int J Biochem Cell Biol.

[43] Brose K, Bland KS, Wang KH, Arnott D, Henzel W (1999). Slit proteins bind Robo receptors and have an evolutionarily conserved role in repulsive axon guidance.. Cell.

[44] Wu W, Wong K, Chen J, Jiang Z, Dupuis S (1999). Directional guidance of neuronal migration in the olfactory system by the protein Slit.. Nature.

[45] Dallol A, Morton D, Maher ER, Latif F (2003). SLIT2 axon guidance molecule is frequently inactivated in colorectal cancer and suppresses growth of colorectal carcinoma cells.. Cancer Research.

[46] Narayan G, Goparaju C, Arias-Pulido H, Kaufmann AM, Schneider A (2006). Promoter hypermethylation-mediated inactivation of multiple Slit-Robo pathway genes in cervical cancer progression.. Mol Cancer.

[47] Dallol A, Krex D, Hesson L, Eng C, Maher ER (2003). Frequent epigenetic inactivation of the SLIT2 gene in gliomas.. Oncogene.

[48] Prasad A, Fernandis AZ, Rao Y, Ganju RK (2004). Slit protein-mediated inhibition of CXCR4-induced chemotactic and chemoinvasive signaling pathways in breast cancer cells.. J Biol Chem.

[49] Kim HK, Zhang H, Li H, Wu TT, Swisher S (2008). Slit2 inhibits growth and metastasis of fibrosarcoma and squamous cell carcinoma.. Neoplasia.

[50] Lazaris AC, Zarogiannos A, Kavantzas N, Zervas A, Giannopoulos A (2006). MLH1 mismatch repair gene product is associated with apoptotic potential of urothelial bladder carcinomas.. Anticancer Res.

[51] Taby R, Issa JP (2010). Cancer epigenetics.. CA Cancer J Clin.

[52] Jin H, Wang X, Ying J, Wong AH, Cui Y (2007). Epigenetic silencing of a Ca(2+)-regulated Ras GTPase-activating protein RASAL defines a new mechanism of Ras activation in human cancers.. Proc Natl Acad Sci U S A.

[53] Seto M, Ohta M, Ikenoue T, Sugimoto T, Asaoka Y (2011). Reduced expression of RAS protein activator like-1 in gastric cancer.. International journal of cancer.

[54] Bos JL, Rehmann H, Wittinghofer A (2007). GEFs and GAPs: critical elements in the control of small G proteins.. Cell.

[55] Bos JL (1989). ras oncogenes in human cancer: a review.. Cancer Res.

[56] Maddox P, Sasieni P, Szarewski A, Anderson M, Hanby A (1999). Differential expression of keratins 10, 17, and 19 in normal cervical epithelium, cervical intraepithelial neoplasia, and cervical carcinoma.. J Clin Pathol.

[57] Ye H, Yu T, Temam S, Ziober BL, Wang J (2008). Transcriptomic dissection of tongue squamous cell carcinoma.. BMC Genomics.

[58] Toyoshima T, Vairaktaris E, Nkenke E, Schlegel KA, Neukam FW (2008). Cytokeratin 17 mRNA expression has potential for diagnostic marker of oral squamous cell carcinoma.. Journal of Cancer Research and Clinical Oncology.

[59] Luo A, Kong J, Hu G, Liew CC, Xiong M (2004). Discovery of Ca2+-relevant and differentiation-associated genes downregulated in esophageal squamous cell carcinoma using cDNA microarray.. Oncogene.

[60] Xu Y, Zhang SZ, Huang CH, Liu XY, Zhong ZH (2009). Keratin 17 identified by proteomic analysis may be involved in tumor angiogenesis.. Bmb Reports.

[61] Regauer S, Reich O (2007). CK17 and p16 expression patterns distinguish (atypical) immature squamous metaplasia from high-grade cervical intraepithelial neoplasia (CIN III).. Histopathology.

[62] Carrilho C, Alberto M, Buane L, David L (2004). Keratins 8, 10, 13, and 17 are useful markers in the diagnosis of human cervix carcinomas.. Hum Pathol.

[63] Wolff EM, Byun HM, Han HF, Sharma S, Nichols PW (2010). Hypomethylation of a LINE-1 promoter activates an alternate transcript of the MET oncogene in bladders with cancer.. PLoS Genet.

[64] Wallenius V, Hisaoka M, Helou K, Levan G, Mandahl N (2000). Overexpression of the hepatocyte growth factor (HGF) receptor (Met) and presence of a truncated and activated intracellular HGF receptor fragment in locally aggressive/malignant human musculoskeletal tumors.. Am J Pathol.

[65] Natali PG, Prat M, Nicotra MR, Bigotti A, Olivero M (1996). Overexpression of the met/HGF receptor in renal cell carcinomas.. Int J Cancer.

[66] Birchmeier C, Birchmeier W, Gherardi E, Vande Woude GF (2003). Met, metastasis, motility and more.. Nat Rev Mol Cell Biol.

[67] Nasser MW, Datta J, Nuovo G, Kutay H, Motiwala T (2008). Down-regulation of micro-RNA-1 (miR-1) in lung cancer. Suppression of tumorigenic property of lung cancer cells and their sensitization to doxorubicin-induced apoptosis by miR-1.. J Biol Chem.

[68] Yan D, Dong Xda E, Chen X, Wang L, Lu C (2009). MicroRNA-1/206 targets c-Met and inhibits rhabdomyosarcoma development.. J Biol Chem.

[69] Nasarre P, Potiron V, Drabkin H, Roche J (2010). Guidance molecules in lung cancer.. Cell Adh Migr.

[70] Karolchik D, Hinrichs AS, Furey TS, Roskin KM, Sugnet CW (2004). The UCSC Table Browser data retrieval tool.. Nucleic Acids Res.

[71] Li H, Ruan J, Durbin R (2008). Mapping short DNA sequencing reads and calling variants using mapping quality scores.. Genome Res.

[72] Audic S, Claverie JM (1997). The significance of digital gene expression profiles.. Genome Res.

[73] Benjamini Y, Hochberg Y (1995). Controlling the false discovery rate: a practical and powerful approach to multiple testing.. Journal of the Royal Statistical Society Series B (Methodological).

[74] Hegedus Z, Zakrzewska A, Agoston VC, Ordas A, Racz P (2009). Deep sequencing of the zebrafish transcriptome response to mycobacterium infection.. Mol Immunol.

[75] Li R, Yu C, Li Y, Lam TW, Yiu SM (2009). SOAP2: an improved ultrafast tool for short read alignment.. Bioinformatics.

[76] Maragkakis M, Reczko M, Simossis VA, Alexiou P, Papadopoulos GL (2009). DIANA-microT web server: elucidating microRNA functions through target prediction.. Nucleic Acids Res.

[77] Friedman RC, Farh KKH, Burge CB, Bartel DP (2009). Most mammalian mRNAs are conserved targets of microRNAs.. Genome Research.

[78] Krek A, Grun D, Poy MN, Wolf R, Rosenberg L (2005). Combinatorial microRNA target predictions.. Nat Genet.

[79] Rajewsky N (2006). microRNA target predictions in animals.. Nat Genet.

[80] Papadopoulos GL, Reczko M, Simossis VA, Sethupathy P, Hatzigeorgiou AG (2009). The database of experimentally supported targets: a functional update of TarBase.. Nucleic Acids Res.

[81] Xiao F, Zuo Z, Cai G, Kang S, Gao X (2009). miRecords: an integrated resource for microRNA-target interactions.. Nucleic Acids Res.

[82] Bindea G, Mlecnik B, Hackl H, Charoentong P, Tosolini M (2009). ClueGO: a Cytoscape plug-in to decipher functionally grouped gene ontology and pathway annotation networks.. Bioinformatics.

