# Supplementary material for: A Systematic Analysis on DNA Methylation and the Expression of Both mRNA and microRNA in Bladder Cancer
Source: PLoS One. 2011 Nov 30;6(11):e28223. doi: 10.1371/journal.pone.0028223 (PMC3227661; doi:10.1371/journal.pone.0028223)
Supplement: Table S10 — Clinical information on the 42 patients sequenced or validated in this study. (DOC) [file pone.0028223.s010.doc]

**Table S10. Clinical information for the 42 patients with bladder** **urothelial carcinoma.**

| **The Patients** | **Age (years)** | **Sex** | **TNM** | **Screening or Validation** |
| --- | --- | --- | --- | --- |
| B4 | 42 | M | T2N0M0 | screening |
| B5 | 53 | M | T4N0M0 | screening |
| B12 | 66 | M | T1N0M0 | screening |
| B13 | 64 | M | T2N0M0 | screening |
| B15 | 40 | M | T4aN0M0 | screening |
| B 16 | 72 | M | T1N0M0 | screening |
| B 20 | 66 | M | T4N0M0 | screening |
| B 30 | 72 | M | T4N0M0 | screening |
| B25 | 65 | M | T4aN0M0 | screening |
| B148 | 51 | M | T4N0M0 | validation |
| B149-5 | 67 | M | T1N0M0 | validation |
| B153 | 75 | M | T1N0M0 | validation |
| B158 | 59 | F | T4N0M0 | validation |
| B169 | 80 | M | T3N0M0 | validation |
| B170 | 48 | M | T2N0M0 | validation |
| B174 | 54 | M | T1N0M0 | validation |
| B176 | 49 | F | T3N0M0 | validation |
| B152 | 60 | M | T1N0M0 | validation |
| B142 | 46 | M | TaN0M0 | validation |
| B154 | 36 | M | T4N0M0 | validation |
| B177 | 73 | M | T1N0M0 | validation |
| B150 | 76 | M | T1N0M0 | validation |
| B151 | 60 | M | T2N0M0 | validation |
| B144 | 59 | M | T3N0M0 | validation |
| B104 | 52 | M | T2N0M0 | validation |
| B105 | 62 | M | T2N0M0 | validation |
| B106 | 56 | M | T2N0M0 | validation |
| B112 | 62 | M | T1N0M0 | validation |
| B115 | 70 | F | T2N0M0 | validation |
| B117 | 36 | M | T2N0M0 | validation |
| B118 | 78 | M | T3N0M0 | validation |
| B119 | 63 | M | T1N0M0 | validation |
| B120 | 52 | M | T1N0M0 | validation |
| B123 | 45 | M | T2N0M0 | validation |
| B124 | 66 | M | T3N0M0 | validation |
| B125 | 52 | M | T1N0M0 | validation |
| B127 | 62 | M | T1N0M0 | validation |
| B128 | 51 | M | T1N0M0 | validation |
| B160 | 56 | M | T1N0M0 | validation |
| B161 | 55 | M | T2N0M0 | validation |
| B166 | 50 | F | T1N0M0 | validation |
| B168 | 60 | M | T3N0M0 | validation |
