# Supplementary material for: A Systematic Analysis on DNA Methylation and the Expression of Both mRNA and microRNA in Bladder Cancer
Source: PLoS One. 2011 Nov 30;6(11):e28223. doi: 10.1371/journal.pone.0028223 (PMC3227661; doi:10.1371/journal.pone.0028223)
Supplement: Table S11 — BSP and RT-qPCR primers used in the validation assays. (DOC) [file pone.0028223.s011.doc]

**Table S11.** BSP and RT**-**qPCR primers used in the validation assays.

| **Gene** | | **BSP Primers (5'-3')** |
| --- | --- | --- |
| ADAMTS9 | | forward primer TAGGAATTGTTTTATTGTTAGGATTTAGTA |
|  |  | reverse primer AACCACTCAACAACAATCTTTCTAC |
| FGFR1 | | forward primer TTTGTTTTTGGGTGGGAGTATT |
|  |  | reverse primer AAAAATAAAAAATTCATTTCCCCTC |
| CRISPLD2 | | forward primer GTGGGGGTTTTAGATTTTGAGAT |
|  |  | reverse primer CCACACAAACATAATTTTCATTAAAC |
| ITPR3 | | forward primer GGGGATTTTTATGTTTTATGGTTTT |
|  |  | reverse primer CCAACCACCTCAAAAATAAACTTATT |
| CACNA1C | | forward primer GAGGTTGTGGGATTTAAGGAGTAG |
|  |  | reverse primer CAATAATCAATTTCCAATCACAACA |
| NFATC1 | | forward primer GGTGTTTTAAGTATAAGAGTTTGGG |
|  |  | reverse primer AACTACAACCATCCACACAAAAAAT |
| TRIP13 | | forward primer GTTGTGTTTATTTGGTTGGTTGTATAA |
|  |  | Right primer ATCCATAACTAAAACCAATTCTCCC |
| PEX14 | | forward primer TATAGAGGTGTTATTTGTTTTTTTT |
|  |  | reverse primer CTCAAAATTCCTACTCCAAACC |
| PNPLA7 | forward primer TTGGTAAGGTTGGTTTTAAATTTTT | |
|  | reverse primer ACATAAAAAACTAAATTTCCCCAAC | |
| ZNF521 | forward primer AGTTATTTTTTGAGAGGTGTGTGTG | |
|  | reverse primer ATAAATCCCCAAAAAAACAAAAAA | |
| MEIS2 | forward primer TTAAATGGTGGAATTAGGATTTTAATTT | |
|  | reverse primer AAATCAAATCAACTATAAAACAACCAAA | |
| NOS2A | forward primer TGGATTTGTAAGTTAAAATTTGGAAA | |
|  | reverse primer CCTATTAACCACCCCAATAAATAATAAA | |
| SLIT2 | | forward primer TTTGTTTGGAGTAGTGGATTTTATT |
|  |  | reverse primer AAAACTACAAAAAAACCCCAAAAAC |
| RASAL1 | | forward primer TATGGGTGTTAGGTATTGTTTTAAG |
|  |  | reverse primer ATTCCAAAACTCTAAATTCCTCATC |
| HIC1 | | forward primer AATTTGGATTATGATATGGTGAGTT |
|  |  | reverse primer CTCCTCCAACTTCTAAAAAAACAAC |
| KRT17 | | forward primer GGAATGTGGTTATGTTTTAGATTGT |
|  |  | reverse primer ATTAAAAAACACTCCCTACCTCAAC |
| **Gene** | **RT-qPCR Primers (5'-3')** | |
| SLIT2 | forward primer AGGTATTCCAAGAGATGTC | |
|  | reverse primer  GTGCTTATTCTGTTGTTACT | |
| RASAL1 | forward primer  ATCTCTGGCACATCTGAC | |
|  | reverse primer   GAGTCTTCTTGATGGTTGAG | |
| HIC1 | forward primer ATATCAGCTTTGACCAAA | |
|  | reverse primer  GAGATTCACAGTGAGAAT | |
| KRT17 | forward primer  CGTGACCAGTATGAGAAG | |
|  | reverse primer  TTCAGTTCCTCTGTCTTG | |
